# Gamified Mobile Computerized Cognitive Behavioral Therapy for Japanese University Students With Depressive Symptoms: Protocol for a Randomized Controlled Trial

**DOI:** 10.2196/15164

**Published:** 2020-04-07

**Authors:** Kengo Yokomitsu, Tomonari Irie, Mayu Sekiguchi, Ayako Shimizu, Hirofumi Matsuoka, Sally Nicola Merry, Karolina Stasiak

**Affiliations:** 1 College of Comprehensive Psychology Ritsumeikan University Osaka Japan; 2 School of Education and Culture Hokusho University Hokkaido Japan; 3 School of Psychological Science Health Sciences University of Hokkaido Hokkaido Japan; 4 HIKARI Lab, Inc Tokyo Japan; 5 School of Dentistry Health Sciences University of Hokkaido Hokkaido Japan; 6 Department of Psychological Medicine University of Auckland Auckland New Zealand

**Keywords:** SPARX, Japan, university students, depressive symptoms

## Abstract

**Background:**

Evidence shows that computerized self-help interventions are effective for reducing symptoms of depression. One such intervention, SPARX, is a gamified mobile computerized cognitive behavioral therapy (cCBT) developed for adolescents in New Zealand, which was shown to be as effective as usual care for young people with mild-to-moderate symptoms of depression. However, gamified cCBT has not yet been tested in Japan.

**Objective:**

This trial is designed to investigate whether a Japanese-adapted version of SPARX improves depressive symptoms in Japanese university students with mild-to-moderate depressive symptoms.

**Methods:**

In this 7-week, multicenter, stratified, parallel-group, superiority randomized trial, participants will be allocated to either a treatment condition (SPARX) or a wait-list control condition. SPARX is a fully automated program, which will be delivered to the mobile phone or tablet device of the participants. SPARX is designed as an interactive fantasy game to guide the user through seven modules that teach key CBT strategies. All participants will be recruited from universities via advertisements on online bulletin boards, the campus newspaper, and posters. Participants in the treatment condition will use the SPARX program weekly. The primary outcome is the reduction of depressive symptoms (using Patient Health Questionnaires-9) measured at baseline and weekly: once after the 7-week intervention and once at a 1-month follow-up. Secondary outcomes include satisfaction with the program and satisfaction with life, measured by the Satisfaction With Life Scale; positive and negative moods, measured by the Profile of Mood States Second Edition; social functioning, measured by the EuroQol Instrument; rumination, measured by the Ruminative Responses Scale; and coping, measured by the Brief Coping Orientation to Problem Experienced Inventory.

**Results:**

This study received funding from The Research Institute of Personalized Health Sciences, Health Sciences University of Hokkaido, and obtained institutional review board approval in September 2019. Data collection began in April 2019.

**Conclusions:**

Results of this trial may provide further evidence for the efficacy of gamified cCBT for the treatment of depression and, specifically, provide support for using SPARX with Japanese university students.

**Trial Registration:**

Japan Primary Registries Network UMIN000034354; https://tinyurl.com/uu7xd77

**International Registered Report Identifier (IRRID):**

DERR1-10.2196/15164

## Introduction

### Background

Depression is a mental health problem commonly experienced by university students. A systematic review examining rates of depression among university students from a range of countries, including North American, European Union, East Asian, and Middle Eastern countries, revealed the weighted mean prevalence rate of depressive disorders of 30.6% [1], which is considerably higher than that reported for the general population (6.6%) [2]. Depression and other mental health concerns such as anxiety and stress negatively impact academic performance [3-5], which may result in students dropping out of university [6] and potentially affect their future career prospects. For example, in Japan, only 1.7% of individuals that dropped out of university reported eventually working as full-time permanent employees for three or more years, whereas 60% of university and graduate school graduates obtained regular employment [7]. Therefore, the mental well-being of tertiary students is not only a health care issue but also an issue that influences the overall socioeconomic welfare of a society.

In Japan, 126 of the 135 universities (93%) have some established support systems to address the mental health needs of university students [8]. However, reaching out to professional mental health services among students is relatively uncommon [9,10] owing to several factors, including skepticism about treatment effectiveness, lack of awareness of help options, and lack of perceived need [11,12]. Therefore, access to university mental health services should be adjusted so that it is both easier and more acceptable [13]. Outside Japan, there have been many trials of internet-based and computer-delivered interventions in recent years, demonstrating significant improvements in depression compared to an inactive control (pooled standardized mean difference –0.43, 95% CI –0.63 to –0.22) [14]. Digital interventions offer important advantages: they are accessible from various locations, they can be a form of outreach to individuals who might not otherwise access traditional face-to-face services [15,16] and are more cost-effective than face-to-face treatment [17]. Moreover, digital interventions can facilitate learning and retention because users can return to the program at their convenience to practice the content more than once [18]. Given that games are familiar and particularly popular with young people in Japan [19,20], game-based interventions have the potential to be engaging and valuable self-help tools to support university students’ mental health [21,22].

In earlier studies, a variety of game-based interventions for mental health suggested potential benefits for mental and physical symptoms [23-25]. At present, the Smart, Positive, Active, Realistic, X-factor thoughts (SPARX) program [26] is the only available game-based intervention developed to specifically deliver cognitive behavioral therapy (CBT) for adolescent users with depressive symptoms [22,27]. SPARX was created, evaluated, and implemented nationally in New Zealand for use by adolescents with mild-to-moderate depressive symptoms [26]. Furthermore, SPARX was used as a depression prevention intervention in a large Australian school trial to reduce depressive symptoms in adolescents prior to final year examinations [28]. In Japan, a smartphone-based CBT program (Kokoro-app) was demonstrated to be effective for depression in a clinical sample [29]; however, there have been no trials of game-based CBT for depression in Japanese university students conducted to date. It is anticipated that SPARX may be well received by university students experiencing depressive symptoms who do not have contact with professional services. However, a formal randomized controlled trial (RCT) is warranted to establish whether SPARX is effective in this population.

### Objectives

The primary objective of this trial is to examine whether SPARX improves depressive symptoms in Japanese university students who present mild-to-moderate symptoms. We hypothesize that SPARX will be more effective for improving depressive symptoms than a wait-list condition. In the systematic review of trials of internet-based and computer-delivered interventions for university students [14], although half of the included trials used an inactive control group (wait-list or no treatment), the other half used an active control (eg, attention training or providing educational information on anxiety and depressive disorders). This will be the first clinical trial of SPARX for university students in Japan. Therefore, we will examine the difference between a SPARX group and a wait-list control group.

The secondary objectives are to determine whether SPARX delivered to Japanese students with mild-to-moderate depressive symptoms leads to (1) enhanced positive mood and reduced negative mood, (2) improved satisfaction with life, (3) improved health-related quality of life, (4) reduced depressive rumination, and (5) improved coping skills.

## Methods

### Study Design

**Figure 1:**
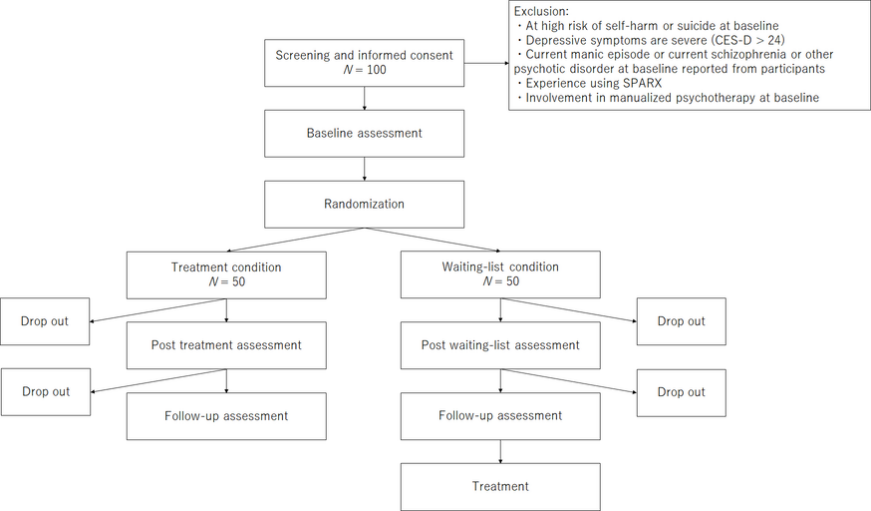
Participant flow of this trial. CES-D: Center for Epidemiological Studies Depression; SPARX: Smart, Positive, Active, Realistic, X-factor thoughts.

This is a multicenter trial and follows a stratified randomized, parallel-group superiority design. Participants will be randomized 1:1 to an intervention arm, which will consist of the game-based computerized CBT program SPARX or a wait-list control condition. Postintervention assessments will be carried out 7 weeks after baseline assessment. The participant flow through the trial is shown in Figure 1.

### Setting

This multicenter study will be conducted at three universities: Health Sciences University of Hokkaido, Hokusho University, and Ritsumeikan University. We plan to recruit participants from April 2019 through March 2020.

### Participants

A total of 100 participants will be sought to take part in this trial. Participants will be included in the trial if they meet the following inclusion criteria at the face-to-face or telephone eligibility interview: (1) mild-to-moderate depressive symptoms on the Center for Epidemiological Studies Depression Scale (CES-D) [30] with scores between 16 and 24 [31,32], (2) age of 18-30 years, (3) attending one of the participating universities, and (4) owning and being able to use a smartphone/tablet device. These inclusion criteria differ from those of the first SPARX study [26]. Namely, we include university students aged 18-30 years instead of high school students, who were recruited for the first SPARX study [26], because when the Japanese version of SPARX was first developed, this app was designed with adult users in mind, including university students, rather than high school students. In addition, we decided to conduct the current study based on the contemporary reality of Japanese university students, because a study conducted by the Ministry of Education, Culture, Sports, Science and Technology of Japan in 2018 [33] found that Japanese university students aged 18-30 years accounted for approximately 99.9% of the total university students in Japan.

Participants will be excluded if they have: (1) high risk of self-harm or suicide at study entry based on a self-report; (2) severe depressive symptoms, as indicated by a CES-D score>24 [31,32]; (3) current manic episodes or current schizophrenia or other psychotic disorders at baseline as reported by a participant; (4) previous experience of using SPARX; and (5) involvement in manualized psychotherapy at baseline. These exclusion criteria are the same as those adopted in the first SPARX study [26] as reference. Merry et al [26] suggested that people exhibiting severe depressive symptoms or at high risk of self-harm or suicide should be excluded because they could not provide a self-help resource as a viable option. In addition, individuals experiencing current manic episodes, schizophrenia, or other psychotic disorders, or are involved in manualized psychotherapy at baseline were excluded because they require medical and psychiatric treatment suitable for their symptoms rather than SPARX or are already receiving suitable psychiatric treatment. If we do not reach the sample size required to test the primary hypothesis of this study, we will extend recruitment to other interested universities and will consider using online recruitment.

### Clinical Safety Procedures

For participants indicating a high risk of self-harm or suicide at screening and at any time during this trial, the researchers will provide information on available mental health care services that the participants can access and will ask the participant to cease participation in this trial. However, participants will be provided with ongoing access to SPARX even if they decide to withdraw from the trial. In relation to ancillary care, the participants will be allowed to contact the researchers at any point during the trial. If any event requiring additional care occurs, a researcher will inform the individual at the contact address or telephone number of the appropriate medical or psychological care and mental health service as provided by the participant’s health insurance.

### Study Procedure

All participants will be recruited from the abovementioned universities via advertisements on online bulletin boards, campus newspapers, and posters. Interested students who contact the researchers via email will be provided with further information related to the study and will be given time to consider their participation. If interested, they will be screened using Google forms (developed by the authors for this study) for eligibility using the Japanese version of the CES-D [30]. The cutoff score of CES-D was verified in Japanese samples [30]. If the score is 16-24, the researcher will continue with the eligibility interview to confirm that all other criteria are met. At the end of the interview, written consent will be obtained. Those who score less than 15 or above 25 will be excluded from the trial but will be provided with free access to SPARX, according to the needs of the participant. Those whose score is 25 or more, indicating severe depressive symptoms [31,32], will be provided with information about available mental health care services.

For participants in the treatment condition, SPARX will be delivered on their mobile phone or tablet device. They will be asked to play the SPARX program weekly at a designated university location (eg, a vacant laboratory or conference room) with one of the researchers (or a research assistant) present in another room (eg, a neighboring laboratory) for the purpose of checking SPARX use and being available to answer any questions from the participant. Each weekly session with SPARX is expected to take about 30 min.

Participants in the wait-list condition group will receive access to SPARX after a 1-month follow-up assessment of the treatment condition group. They will be asked to fill out assessments at the same time as the assessments of the treatment condition group at experimental laboratories, in conference rooms at each university, or on online Google forms. They will not be granted access to SPARX until all assessments have been completed.

To address potential issues of contamination, we forbid participants from talking about any theme of SPARX, such as the use of SPARX and any impressions of this study, during trial participation because other students in their university campus are participating in the current trial.

### Ethics

This study protocol was reviewed and approved by the institutional review board of Hokusho University (2017-021; February 20, 2018), Ritsumeikan University (2018-028; September 19, 2018), and Health Sciences University of Hokkaido (18-006, December 8, 2018). Furthermore, this study has been registered with UMIN (000034354). Details of this trial will be explained by the researchers to the participants before study participation. Written informed consent will be obtained from participants prior to entering them into the trial. All outcome data, including written informed consent, will be securely stored at the participating university (eg, Ritsumeikan University) in a locked cabinet. All identifying information will be removed, and the participants will only be identified by a unique participant number.

### Randomization

Participants (N=100) who fulfil all eligibility criteria will be randomized at a 1:1 ratio to either the SPARX intervention group or the wait-list control condition. The randomization sequence will be generated by the trial statistician and stratified by site. The final randomization lists will be computer-generated and concealed in a secure study database until the end of data collection/data lock.

### Sample Size

A meta-analysis of RCTs examining the effects of CBT in comparison to wait-list on depression in university students showed an effect size of 1.13 [34]. However, the effect sizes were smaller (0.56 [35] and 0.64 [36]) for studies in adults compared to those of internet-based and other computerized psychological treatments with wait lists. A meta-analysis of RCTs to examine the effectiveness of game-based digital interventions showed an overall effect size of 0.66 (against a wait-list control condition), 0.47 (with a target population of adolescent users), and 0.41 (type of game: psychoeducation and training, including the SPARX study) [22]. Additionally, a meta-analysis of RCTs to examine the effectiveness of technology-delivered interventions for depression and anxiety in children and adolescents showed an effect size of 0.45 compared to the wait-list group [37]. Considering these findings, we assumed a similar effect size (0.60) to that of internet-based and other computerized psychological treatments or game-based digital interventions against wait-list [22,35,36] for the reduction of primary outcomes assessed using Patient Health Questionnaire-9 (PHQ-9).

A power analysis showed that a target sample size of 90 was needed to detect an effect size of 0.60 with alpha=.05 and a power of 0.80 for a two-tailed test. Considering an anticipated 10% dropout [38], we will aim to recruit at least 100 (50 per group) participants to test the primary hypothesis of this study.

### Intervention

The treatment condition in this study consists of a game-based computerized CBT program called SPARX. It takes the form of an interactive fantasy game designed to take the user through seven modules that teach key CBT strategies. When the user begins SPARX, they meet the character of the Guide who introduces them to the “game world” and the challenges ahead, after which the user selects their avatar and begins the quest. Each module finishes with the avatar returning to the Guide who then explains how the “game skills” can be used in “real life”. The first module includes brief psychoeducation related to depression, with a brief introduction of relaxation (slow and controlled breathing) and hope. The remaining modules cover the following: activity scheduling/behavioral activation, interpersonal skills (communication, assertiveness, and negotiation), problem solving, cognitive restructuring (identifying common cognitive distortions and challenging negative thoughts), mindfulness, and relapse prevention (Textbox 1). Each module is designed to take about 20-30 min and the user is encouraged to practice the skills in the interim (the equivalent of homework).

**Outline of content and core skills covered in each level of Smart, Positive, Active, Realistic, X-factor thoughts (SPARX).**

- Level 1 (finding hope): psychoeducation about depression, introduction to the cognitive behavioral therapy model, introducing gloomy negative automatic thoughts and “hope” (people recover from depression), relaxation (controlled breathing)
- Level 2 (being active): activity scheduling, behavioral activation, relaxation (progressive muscle relaxation), basic communication, and interpersonal skills
- Level 3 (dealing with emotions): dealing with anger and hurt feelings, interpersonal skills (assertiveness, listening, and negotiation)
- Level 4 (overcoming problems): problem solving, cognitive restructuring (identifying smart, positive, active, realistic, X-factor thoughts)
- Level 5 (recognizing unhelpful thoughts): cognitive restructuring (recognizing different types of gloomy negative automatic thoughts)
- Level 6 (challenging unhelpful thoughts): cognitive restructuring (learning to challenge or swap negative thoughts for helpful ones), interpersonal skills continued (negotiation skills)
- Level 7 (bringing it all together): recap of all skills, mindfulness (tolerating distress), relapse prevention

**Figure 2:**
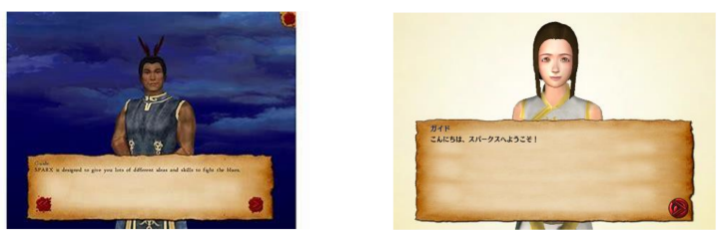
Guide characters in the SPARX original (left) and Japanese (right) versions.

The Japanese version of SPARX was developed by the Japanese companies HIKARI Lab, Inc, and SmileBoom Co, Ltd, with approval of the developers of the original version. The original English language was translated into Japanese by the fourth author (AS) who has a master’s degree in clinical psychology and is a native Japanese speaker and a fluent speaker of English. A key difference between the original and Japanese versions was the change of the Guide character’s gender from male to female. In the original version, the Guide was male, stylized to look like a Maori (the indigenous people of New Zealand) chief to give a powerful impression. However, the developers in the Japanese version were concerned that this might not suit Japanese audiences and instead adopted a female character wearing a white national costume, with a more gentle and maternal expression (Figure 2). Other modifications include the following: for all SPARX characters, the face and eyes were slightly rounded and the color schemes were made brighter to be more reminiscent of the Japanese anime esthetic; login and menu screens were slightly changed to facilitate use; by using a notification function, the user can obtain information related to mental health once a week; how to process SPARX is displayed; and as the user plays the game and proceeds through the stages, the sky color becomes brighter. There were also some necessary content changes to make the program more applicable to Japanese adult and university students as users (eg, original version: assignment/homework anxiety; Japanese version: work presentation anxiety). With the exception of these minor and complementary fixes in developing the Japanese version of SPARX, no major changes to the original were made, such as a change of the main story of SPARX. The original version was delivered on a user’s personal computer, whereas the Japanese version is delivered on a mobile phone or tablet device.

### Wait-List Control

Participants randomized to the control group will be placed on a wait list for 11 weeks; that is, until after the 1-month follow-up assessment of the group assigned to the intervention. The participants on the wait list will then be offered the opportunity to take part in the SPARX program in the same way as those in the intervention group.

### Measures

The primary outcome (Japanese version of PHQ-9) will be measured at baseline (T1), weekly throughout the interventions (T2-T7), at postassessment 1 week after the 7 intervention weeks (T8), and at 1-month follow-up (T9). Other assessments will be conducted at preassessment (T1), postassessment (T8), and 1-month follow-up (T9). Table 1 shows the schedule of assessments at various time points. At baseline (T1), demographics data will be collected, including gender, age, major in university, year of study, residence status; presence or absence of visits to psychiatric and counseling services; and presence or absence of the use of a gamified psychological program.

**Table 1:** Overview of measurements.

Outcome	Scale	T1^a^	T2-T7^b^	T8^c^	T9^d^
Demographics	Developed by the authors	✓			
Depressive symptoms	PHQ-9^e^	✓	✓	✓	✓
Satisfaction with life	SWLS^f^	✓		✓	✓
Negative and positive moods	POMS-2^g^	✓		✓	✓
Social functions	EQ-5D^h^	✓		✓	✓
Rumination	RSS^i^	✓		✓	✓
Coping	Brief COPE^j^	✓		✓	✓
Stressor	DLSS^k^	✓		✓	✓
Satisfaction and acceptability	Developed by the authors				✓

^a^T1: baseline assessment (preassessment).

^b^T2-T7: assessment at each level.

^c^T8: assessment 1 week after the program.

^d^T9: assessment at 1-month follow-up.

^e^PHQ-9: Japanese version of Patient Health Questionnaire-9.

^f^SWLS: Satisfaction With Life Scale.

^g^POMS-2: Profile of Mood Scale, second edition.

^h^EQ-5D: Japanese EuroQol instrument.

^i^RSS: Japanese version of the Ruminative Responses Scale.

^j^Brief COPE: Brief Coping Orientation to Problem Experienced inventory.

^k^DLSS: Daily Life Stress Scale for undergraduates.

#### Primary Outcome Measure

The primary outcome measure is the Japanese version of PHQ-9. The PHQ-9 is a self-reported questionnaire for assessing depressive symptoms of the preceding 2 weeks [39]. The questionnaire consists of nine items evaluated using a 4-point Likert scale to indicate the extent to which the participant agrees with the values expressed in each item (0, not at all; 3, nearly every day). Higher scores indicate more severe depressive symptoms. In an earlier study [39], this scale demonstrated good internal consistency (alpha=.93) and good convergent validity (correlation coefficient with the Kessler Psychological Distress Scale: *r*=.81) in a Japanese clinical population.

#### Secondary Outcome Measures

The following sections will discuss the secondary measures used.

##### Japanese Version of the Profile of Mood States Second Edition (POMS-2)

This is a self-reported questionnaire for assessing positive and negative moods during the preceding week [40]. The questionnaire consists of 35 items evaluated on a 5-point Likert scale (0-4). For this study, we will use total mood disturbance; higher scores indicate more negative mood states. The reliability and validity of POMS-2 for a Japanese population have been reported [40].

##### The Satisfaction With Life Scale (SWLS)

This is a self-reported questionnaire for assessing satisfaction with life [41]. The questionnaire consists of five items evaluated on a 7-point Likert-type scale (1-7). Higher scores indicate more satisfaction with life. Its reliability and validity for a Japanese population have been reported [41].

##### The Japanese EuroQol Instrument (EuroQol)

This is a self-reported questionnaire for assessing health-related quality of life [42]. The questionnaire consists of five items evaluated on a 5-point Likert-type scale (1-5). Higher scores indicate worse health-related quality of life. Its reliability and validity for a Japanese population have been reported [43].

##### The Japanese Version of the Ruminative Responses Scale (RSS)

This is a self-reported questionnaire for assessing the frequency of depressive rumination [44]. The questionnaire consists of 22 items evaluated on a 7-point Likert-type scale (1-7). Higher scores indicate more frequent depressive rumination. Its reliability and validity for a Japanese population have been reported [45].

##### The Brief Coping Orientation to Problem Experienced Inventory (Brief COPE)

This is a self-rated questionnaire for assessing coping skills [46]. The questionnaire consists of 28 items evaluated on a 4-point Likert-type scale (1-4). Higher scores represent high levels of coping skills. Its reliability and validity for a Japanese population have been reported [47].

##### The Daily Life Stress Scale for Undergraduates (DLSS)

This is a self-reported questionnaire for assessing daily life stressors for undergraduates [48]. The questionnaire consists of 23 items evaluated on a 5-point Likert-type scale (1-5). Higher scores indicate more frequent stressors. Its reliability and validity for a Japanese population have been reported [48].

The final secondary outcome will be a measure of the satisfaction and acceptability of the intervention. All participants will be asked to rate four statements related to the perceived helpfulness, satisfaction with use, and the depth of understanding of the content of SPARX on an 11-point scale (0-10). Participants will also be able to write free text comments. The following questions will be asked: “How useful was this program for you [0 (not at all), 10 (very useful)]? Specifically, which components of this program were useful for you (write free text comments)?” “How satisfied are you with this program [0 (not at all), 10 (very satisfied)]?” “Please tell me about the pros and cons of this program (write free text comments)?” “How fun was this program for you [0 (not at all), 10 (very fun)]?” These items were developed specifically for the current trial.

### Statistical Analyses

The analysis for primary outcome measures will be conducted using a linear mixed model (LMM). The LMM approach was selected because of its strength in accommodating missing data and its ability to incorporate random effects into analyses. In these analyses, the dependent variable is the PHQ-9 score, and the independent variables are assignment (treatment condition, wait-list condition) and time [preassessment (T1), each session assessment (T2-T7), post-assessment (T8), and follow-up assessment (T9)], with interaction of the assignment and time as fixed-effect variables and participants as a random-effects variable. Secondary outcomes (outcome measures, process measures, and evaluation of the program) will be analyzed in the same way as the primary outcome. Subgroup analyses are not planned. For all analyses, *P*<.05 will be inferred as statistically significant. Up-to-date versions of R software (R Foundation for Statistical Computing, Vienna, Austria) will be used to analyze the quantitative data.

In the satisfaction/acceptability measure, free comments will be analyzed using thematic analysis.

## Results

The primary outcome is the presence of depressive symptoms at 1-month follow-up. Several secondary outcomes will be measured, such as positive and negative mood, satisfaction with life, health-related quality of life, depressive rumination, and coping skills. In addition, satisfaction and acceptability of the intervention will be measured, such as the perceived helpfulness, satisfaction with use, and the depth of understanding of the content of SPARX. Dropout rates will be measured to study the feasibility of SPARX.

This study received funding from The Research Institute of Personalized Health Sciences, Health Sciences University of Hokkaido, and obtained institutional review board approval in September 2019. Data collection began in April 2019. Study enrollment is still ongoing. As of April 2019, 60 subjects have been recruited and baseline data were collected. Moreover, 24 subjects have already been allocated to the treatment group and have begun the intervention. We expect to complete the study by March 2020, followed by data analysis and submission of the final report by the end of 2020.

Irrespective of the results, the results of this trial will be published in a peer-reviewed journal and communicated through presentations at national and international conferences. Study participants will be informed about the trial results via a plain-language summary of the results that will be sent to them. Academic papers and summary reports will be provided to the funders.

## Discussion

SPARX is a game-based intervention that was developed to deliver CBT for adolescents with depressive symptoms [22,27]. This program has been demonstrated to be effective for high school students with mild-to-moderate symptoms of depression in New Zealand [26]. The aim of this trial is to investigate the efficacy of the program in Japanese university students using a superiority RCT comparing SPARX with a wait-list control. The findings of this trial are expected to add to evidence of the efficacy of the game-based computerized CBT for young people with depressive symptoms. These results will provide important knowledge for implementation of computerized CBT into existing health support services for university students in the future. However, in this trial, for the purpose of checking SPARX use and being able to answer any questions from a participant, participants in the treatment condition play the SPARX program at a designated university location with one of the researchers present in another room. Therefore, we are not examining the pure effect of SPARX as a self-help tool. This procedure could influence the intervention effect or dropout ratio. In addition, to clarify the benefits and limitations of the effect of SPARX on the university students' depressive symptoms, the effects of SPARX in comparison to an active control will need to be assessed in the future.

## Appendix

Multimedia Appendix 1 CONSORT-eHEALTH checklist (V 1.6.1).

## Abbreviations

Brief COPE: Brief Coping Orientation to Problem Experienced inventory
CBT: cognitive behavioral therapy
CES-D: Center for Epidemiological Studies Depression
DLSS: Daily Life Stress Scale for undergraduates
EuroQol: EuroQol Instrument
LMM: linear mixed model
PHQ-9: Patient Health Questionnaire-9
POMS-2: Profile of Mood States Second Edition
RSS: Ruminative Responses Scale
SPARX: Smart, Positive, Active, Realistic, X-factor thoughts
SWLS: Satisfaction With Life Scale
UMIN: University Hospital Medical Information Network
